# Anti-symmetric Compton scattering in LiNiPO _4_: Towards a direct probe of the magneto-electric multipole moment

**DOI:** 10.12688/openreseurope.13863.2

**Published:** 2022-05-05

**Authors:** Sayantika Bhowal, Daniel O'Neill, Michael Fechner, Nicola A. Spaldin, Urs Staub, Jon Duffy, Stephen P. Collins

**Affiliations:** 1 Materials Theory, ETH Zürich, Zurich, 8093, Switzerland; 2 Department of Physics, University of Warwick, Coventry, CV4 7AL, UK; 3 Condensed Matter Dynamics Department, Max Planck Institute for the Structure and Dynamics of Matter, Hamburg, 22761, Germany; 4 Swiss Light Source, Paul Scherrer Institute, Villigen, 5232, Switzerland; 5 Diamond Light Source, Didcot, Oxfordshire, OX11 0DE, UK

**Keywords:** Magneto-electric toroidal moment, Compton scattering, Density functional theory, Lithium transition metal phosphate

## Abstract

Background: Magnetoelectric multipoles, which break both space-inversion and time-reversal symmetries, play an important role in the magnetoelectric response of a material. Motivated by uncovering the underlying fundamental physics of the magnetoelectric multipoles and the possible technological applications of magnetoelectric materials, understanding as well as detecting such magnetoelectric multipoles has become an active area of research in condensed matter physics. Here we employ the well-established Compton scattering effect as a possible probe for the magnetoelectric toroidal moments in LiNiPO _4_.

Methods: We employ combined theoretical and experimental techniques to compute as well as detect the antisymmetric Compton profile in LiNiPO _4_. For the theoretical investigation we use density functional theory to compute the anti-symmetric part of the Compton profile for the magnetic and structural ground state of LiNiPO _4_. For the experimental verification, we measure the Compton signals for a single magnetoelectric domain sample of LiNiPO _4_, and then again for the same sample with its magnetoelectric domain reversed. We then take the difference between these two measured signals to extract the antisymmetric Compton profile in LiNiPO _4_.

Results: Our theoretical calculations indicate an antisymmetric Compton profile in the direction of the t _y _toroidal moment in momentum space, with the computed antisymmetric profile around four orders of magnitude smaller than the total profile. The difference signal that we measure is consistent with the computed profile, but of the same order of magnitude as the statistical errors and systematic uncertainties of the experiment.

Conclusions: While the weak difference signal in the measurements prevents an unambiguous determination of the antisymmetric Compton profile in LiNiPO _4_, our results motivate  further theoretical work to understand the factors that influence the size of the antisymmetric Compton profile, and to identify materials exhibiting larger effects.

## I. Plain language summary

Materials containing magnetic dipoles, made from the north and south poles of magnets, have been used for millennia for navigation (since they line up along the earth’s magnetic fields), and in modern times for data storage (with their orientations representing the 1s and 0s of digital electronics). Analogous to these magnetic dipoles with their north and south poles are electric dipoles, made of positive and negative charges. Materials containing both electric and magnetic dipoles are rather rare and are an active area of research both because of their potential technological applications in memory, logic or sensor components and because of their intriguing fundamental physics. In particular, the basic electro-magnetic building blocks of such materials, made of composites of electric and magnetic dipoles, are of huge interest, and are likely related to exotic behaviors such as superconductivity (the conduction of electricity with no resistance) and other unusual electrical and magnetic properties. In this research, we describe an advanced experimental technique for detecting one of these composites – the so-called magnetoelectric multipole moment – using high-energy x-ray beams.

## II. Introduction

Magnetoelectric (ME) multipoles are key to understanding the linear ME response in solids, in which an applied electric field induces a linear order magnetization, and vice versa. In particular, the second-rank ME multipole tensor, defined as $M_{ij}=\int r_{i}\mu_{j}(\vec{r})d^{3}r$ ^1, 2^, where $\vec{\mu }$ ( $\vec{r}$ ) is the magnetization density, has the same symmetry as the linear ME response tensor. Both are only non-zero when space-inversion ( *I*) and time-reversal ( *T*) symmetries are broken simultaneously, and there is a one-to-one correlation between their components. For example, materials with an antisymmetric off-diagonal linear ME response also have non-zero antisymmetric off-diagonal elements in their *M _ij_* tensor ^3^.

The ME multipole *M _ij_* tensor can be decomposed into three irreducible (IR) components, the ME monopole, ME dipole (toroidal) moment, and ME quadrupole moment, as summarized in Table I ^1, 2^. In the present work, we are particularly interested in the ME toroidal moment $\vec{t}=\frac{1}{2}\int \vec{r}\times \vec{\mu }(\vec{r})d^{3}r,$ the components of which form the antisymmetric off-diagonal elements of the multipole tensor *M _ij_*,

**Table I.** The three irreducible components of the magnetoelectric (ME) multipole tensor *M _ij_*, that contribute to the second order term $\varepsilon_{int}^{\left(2\right)}$ in the multipole expansion of the interaction energy in presence of an external magnetic field $\vec{H}$ .

ME multipole	ME monopole ( *a*)	ME toroidal moment ( $\vec{t}$ )	ME quadrupole moment ( *q _ij_*)
Definition	$a=\frac{1}{3}M_{ii}=\frac{1}{3}\int \vec{r}\cdot \vec{\mu }(\vec{r})d^{3}r$	$t_{i}=\frac{1}{2}\varepsilon_{ijk}M_{jk}=\frac{1}{2}\int \vec{r}\times \vec{\mu }(\vec{r})d^{3}r$	$q_{ij}=\frac{1}{2}\int \left(r_{i}\mu_{j}+r_{j}\mu_{i}-\frac{2}{3}\delta_{ij}\vec{r}\cdot \vec{\mu }\right)d^{3}r$
Rank	0 (scalar)	1 (vector)	2 (symmetric traceless tensor)
$\varepsilon_{int}^{\left(2\right)}=-\int r_{i}\mu_{j}(\vec{r})\;\partial_{i}H_{j}(0)d^{3}r$	$-a{\left(\vec{\nabla }\cdot \vec{H}\right)}_{\vec{r}=0}$	$-\vec{t}\cdot {\left(\vec{\nabla }\times \vec{H}\right)}_{\vec{r}=0}$	− *q _ij_* ( *∂ _i_H _j_* + *∂ _j_H _i_*)

$$t_{i}=\frac{1}{2}\varepsilon_{ijk}M_{jk}\;.\;(1)$$

The ME toroidal moment has been proposed as the order parameter for a form of hidden ferroic order, known as *ferrotoroidic*, to complete the set of primary ferroics with the existing established ferromagnetism, ferroelectricity, and ferroelasticity ^1, 4, 5^. This proposal motivated considerable interest in ME toroidal moments in solids, leading to experimental efforts to detect them using resonant x-ray diffraction ^6– 10^, magneto chiral dichroism ^11, 12^, and optical measurements ^13^, as well as to image ferrotoroidic domains ^5^. While a theory of *toroidization* (toroidal moment per unit volume) in periodic solids has been developed ^14^, a direct and quantifiable link of detected signals from toroidal moments to the underlying electronic structure is still lacking.

Recently, the occurrence of an antisymmetric component in the Compton scattering profile, which measures the electron momentum density *ρ*( $\vec{p}$ ), was proposed as a possible direct probe of the ME toroidal moment ^15^. For materials that are symmetric in either or both of *I* and *T*, the Compton profile, *J*( *p*), which is a projection of electron momentum density of the form *J*( *p _z_*) = *∫* *ρ*( $\vec{p}$ ) *dp _x_dp _y_*, is symmetric in momentum space, because under both *T* and *I*, $\vec{p}$ → – $\vec{p}$ . For materials that lack *both I* and *T* symmetries, however, an antisymmetric contribution to the Compton profile is allowed, suggesting Compton scattering as a sensitive probe of atomic-scale magnetoelectric properties. A first investigation was made using magnetoelectric GaFeO _3_^15^, for which a density functional study of the ideal material predicted a measurable asymmetry. The measured Compton asymmetry, however, was within the experimental uncertainty, possibly due to the well-known inter-site mixing of Ga and Fe in GaFeO _3_, and so no clear assignment of a toroidal moment could be made.

Here, we present a combined theoretical and experimental Compton scattering study of a magnetoelectric material, lithium nickel phosphate, that has no reported tendency to site disorder. LiNiPO _4_ is an anti-ferromagnetic insulator, which shows an off-diagonal linear ME response ^16– 18^ and hosts a ME toroidal moment ^2^ in its magnetic ground state. (Note that many magnetic-field induced magnetic transitions have recently been identified, resulting in other phases with linear and quadratic ME effects which we do not treat here ^18^.) Our calculations using density functional theory (DFT) indeed show the presence of an antisymmetric Compton profile along the *y* direction in momentum space, consistent with the presence of the ME toroidal moment component *t _y_* in LiNiPO _4_. The calculated intensity of the antisymmetric part of the profile is about four orders of magnitude smaller than that of the corresponding total profile. Our measured antisymmetric signal is of the same order of magnitude as our computed value, but is also of the same order of magnitude as the statistical errors and systematic uncertainties of the experiments. Our main finding, therefore, is that, while an antisymmetric Compton scattering would indeed indicate the existence of a magnetoelectric multipole in a material, improvements in the experimental sensitivity and/or identification of materials with a larger response will be necessary for an unambiguous determination.

We organize the paper as follows. In section III, we provide a detailed description of the crystal structure of LiNiPO _4_ and discuss the theoretical and experimental methods used in the present work to analyze its Compton profile. This is followed by the discussion of our results in section IV. Here, we first discuss the density functional results and the experimental results individually and, then, compare our theory and experiments in order to gain insight into the measured Compton profile in LiNiPO _4_. We also discuss the possible roles of domain averaging and convolution of the profile in reducing the antisymmetric signal in the measurements. Finally, we summarize our findings in section V.

## III. Methods

In this section, we discuss the crystal structure of LiNiPO _4_, followed by the theoretical and experimental methods that were employed in the present work to obtain and study the Compton profile of the material.

### A. Crystal structure

**Figure 1.**
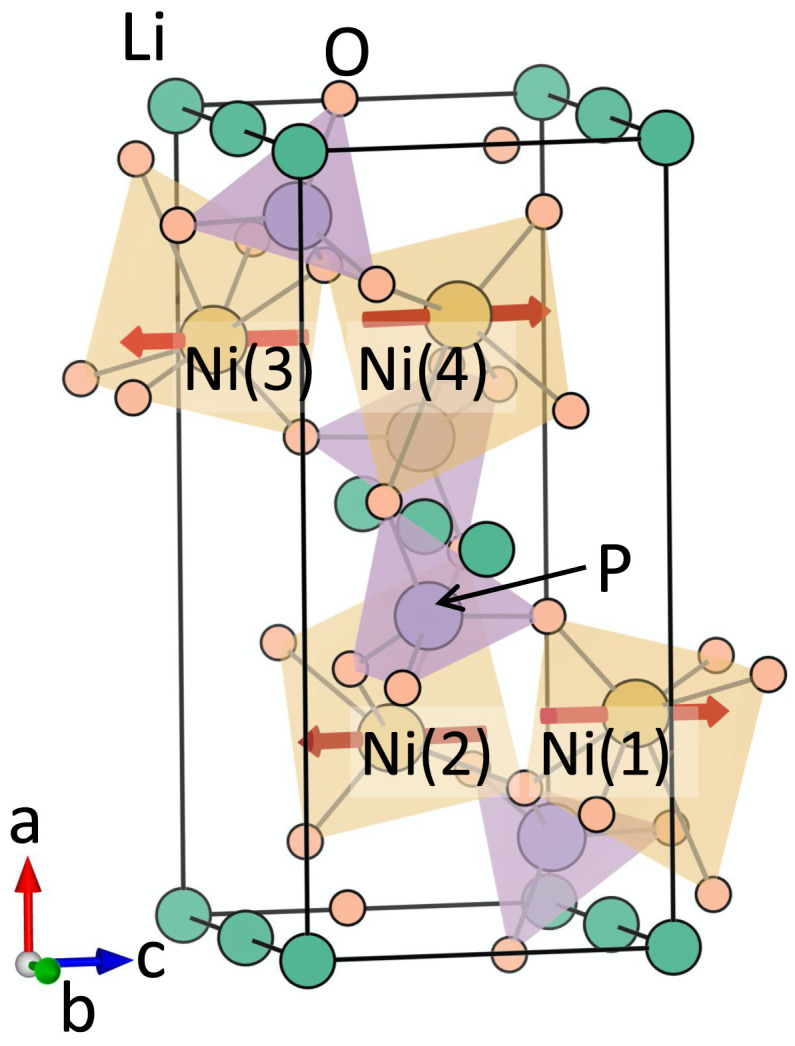
The crystal and magnetic structure of LiNiPO _4_. The red arrows denote the spin moment at the Ni sites corresponding to the magnetic ground state with *Pnm′a* symmetry.

LiNiPO _4_ crystallizes in the orthorhombic *Pnma* structure, with the point group symmetry *D*_2 *h*_^19^. The unit-cell structure is shown in Figure 1. As seen from this figure, the crystal structure consists of distorted NiO _6_ octahedra, which are connected to each other by PO _4_ tetrahedral units. The unit cell consists of four formula units, with the four Ni atoms occupying the Wyckoff positions 4 *c*. In contrast to the previously studied GaFeO _3_, the crystal structure of LiNiPO _4_ has inversion *I* symmetry in the absence of magnetic ordering. The magnetic arrangement at the Ni sites in the ground state breaks both *I* and time-reversal *T* symmetries, leading to a linear ME effect and a non-zero toroidal moment. The combined *IT* symmetry remains preserved in this magnetic ground state.

### B. Computational methods

The electronic structure, the antisymmetric Compton profile, and the ME multipoles for LiNiPO _4_ are obtained using the linearized augmented plane wave (LAPW) method of density functional theory as implemented in the ELK code (version: 4.0.15) ^20, 21^. Within the density functional theory method, the single particle Kohn-Sham equations are solved to obtain the band energies, which are used later to compute the electron momentum density. Spin-orbit coupling (SOC) is included explicitly in the calculations. A local Hubbard *U* correction of *U*_eff_ = *U* − *J* = 4.25 eV is applied to the Ni 3 *d* electrons, within the LDA+SOC+ *U* formalism. We use the default ELK atomic species files in calculations, treating Li: 1 *s* ^2^2 *s* ^1^, Ni: 3 *p* ^6^3 *d* ^8^4 *s* ^2^, P: 3 *s* ^2^3 *p* ^3^, and O : 2 *s* ^2^2 *p* ^4^ electrons as valence electrons. The corresponding muffin-tin radii for Li, Ni, P, and O are taken to be 2.0, 2.4, 2.2, and 1.8 a.u. respectively. In order to achieve self consistency, we use a basis set of *l _max_*_( *apw*)_ = 8, we sample the Brillouin zone with a 3 × 6 × 6 k-point mesh, and take the product of the muffin-tin radius and the maximum reciprocal lattice vector to be 7. The calculations are carried out using the reported relaxed atomic positions calculated in LiNiPO _4_^2^.

Once self-consistency is achieved, we compute the electron momentum density using the same ELK code (version: 4.0.15). This is further projected onto the selected momentum directions ( $\vec{p}$ ) in order to obtain the desired Compton profile *J*( $\vec{p}$ ) ^21^. Next, we separate out the computed profile into symmetric *J ^s^* ( $\vec{p}$ ) and antisymmetric *J ^a^* ( $\vec{p}$ ) parts using the following relation,

$$\begin{array}{l} J\left(\vec{p}\right)=2^{-1}[J\left(\vec{p}\right)+J\left(-\vec{p}\right)]+2^{-1}[J\left(\vec{p}\right)-J\left(-\vec{p}\right)]\\ \;=J^{s}\left(\vec{p}\right)+J^{a}\left(\vec{p}\right) \end{array}\;(2)$$

Additional calculations with a denser 5 × 10 × 10 k-point mesh confirm the convergence of each of the parts, *J ^s^* ( $\vec{p}$ ) and *J ^a^* ( $\vec{p}$ ). We normalize both profiles to the total number of valence electrons per formula unit of LiNiPO _4_, 48, and add the isotropic core contribution to the symmetric part to obtain the total calculated Compton profile, which should be comparable with the measured profile. The core contribution is obtained from the Hartree-Fock calculations of Biggs *et al.* ^22^. To compare the computed antisymmetric Compton profile with the measured value, we further convolute the computed profile with a Gaussian of full-width at half maximum (FWHM) = 0.44 a.u. to mimic the experimental momentum resolution, which is dominated by the detector energy resolution of Δ *E/E* ~ 7 × 10 ^−3^.

We calculate the ME multipoles in the sphere around the Ni sites (referred to as the atomic-site contributions in Refs. 2 and 14) by decomposing the density matrix *ρlm,l′m′* as described in Ref. 2. The parity-odd tensor moments have contributions only from the odd *l* – *l′* terms, i.e., *l* must be different from *l′*; for the case of Ni with its valence *s*, *p* and *d* electrons, this means that we include the *p* − *s* and *p* − *d* matrix element contributions, which provide the measure of the multipoles. Note that the computed multipoles are in *µ _B_* unit and we label them as ${\tilde{t}}_{y},$ $\tilde{q}$ *_xz_*, etc. in Table II.

**Table II.** The computed magnitudes and relative signs of magnetoelectric (ME) multipoles and magnetic moments at the Ni site (Wyckoff position 4c) corresponding to the *Pn* $m^{\prime }$ *a* magnetic structure. The Ni(1), Ni(2), Ni(3), and Ni(4) atoms are indicated in Figure 1.

ME multipoles	Magnitude (10 ^–3^ *µB*)	Relative orientation at Ni(1)-Ni(2)-Ni(3)-Ni(4)	Magnetic moment	Magnitude *µB*	Relative orientation at Ni(1)-Ni(2)-Ni(3)-Ni(4)
${\tilde{t}}_{y}$	5.1	- - - -	*m _x_*	0.01	+ + - -
$\tilde{q}$ *_xz_*	8.2	- - - -	*m _y_*	0	
$\tilde{a}$	5.7	+ - + -	*m _z_*	1.69	+ - - +
$\tilde{q}$ _*x* ^2^− *y* ^2^_	0.3	+ - + -			
$\tilde{q}$ _*z* ^2^_	5.9	+ - + -			

### C. Experimental techniques

**Figure 2.**
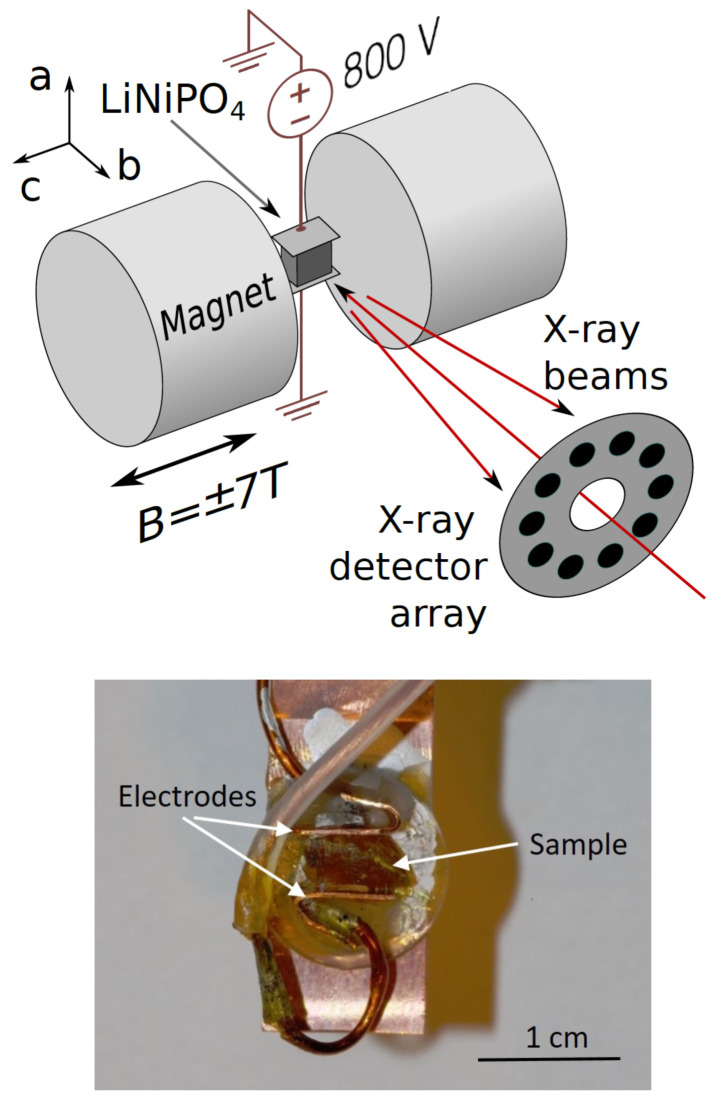
Experimental set up used to measure the antisymmetric Compton profile in LiNiPO _4_. The upper panel shows a schematic of the experimental set up, with *a*, *b* and *c* indicating the orientations of the crystal axes. The polar *a* axis was oriented vertically in the setup. A reversible magnetic field was applied along the crystal *c* axis. The *b* axis, which is the orientation of the *t _y_* toroidal moment, was aligned close to the direction of photon momentum transfer, which defines the projection direction for the electron momentum density. The sample temperature was maintained at T~10K by the variable temperature stage of a helium-cooled superconducting magnet. The incident, linearly polarized x-ray beam of energy 184.3 keV passed through the aperture of a 10-element Ge detector, which subsequently measured the energy spectrum of the Compton scattered x-rays close to back-scattering. The lower panel is a photograph of the crystal on the sample holder with electrodes attached perpendicular to the *a*-axis (vertical in the picture).

Our experiment, carried out on BL08W(A) at SPring-8, utilized the Warwick University superconducting magnet to provide a 7T field perpendicular to the beam, coupled with a high voltage (800V), applied perpendicular to the beam and magnetic field. The experimental set up is shown schematically in Figure 2. The sample was warmed to above 25 K (well above the ordering temperature of 20.8 K), before being cooled through the ordering phase transition to around T=12 K, while applying the magnetic and electric fields simultaneously. Then the cycle was repeated in an + - - + magnetic field sequence, with the same electric field but opposite magnetic field. Field cooling with crossed electric and magnetic fields in this geometry should produce a single magnetoelectric domain state with its net magnetoelectric toroidal moment along the beam direction, as required for our experiment. Any incomplete domain alignment would result in a reduction in the signal.

During the experiment, the greatest practical challenge was arcing due to the high voltage and low pressure helium exchange gas environment. We were therefore only able to provide a high enough electric field for one polarity and with the magnetic field applied. We were then able to reverse the magnetoelectric domains by ramping the magnetic field at ≥ 25 K, although this required long acquisition times of one hour per cycle period. During this time even small drifts in detector gain can lead to parasitic signals that had to be treated very carefully.

The energy spectrum of the measured inelastic scattering signal is, in general, easily mapped onto momentum space since the double differential cross-section is almost directly proportional to the Compton profile *J*( *p _z_*) (see, for example, Ref. 23). Here, we were interested primarily in the antisymmetric difference profile, obtained by subtracting data sets measured with reversed magnetoelectric domains. Since there is a symmetry requirement for the two profiles to have the same area, the datasets were normalized prior to subtraction to ensure that this requirement was satisfied.

## IV. Results and discussion

### A. Density-functional theory results

Our calculated lowest-energy magnetic structure of LiNiPO _4_ has magnetic space group *Pnm′a*, consistent with experimental measurements. In this magnetic configuration, the magnetic moments at the Ni sites are anti-ferromagnetically arranged, with computed spin moment of 1.69 *µ _B_* at each Ni site and zero net magnetization. The Ni spins are primarily oriented along the *z* direction with a small component along the *x* direction at each site, as a result of a small antiferromagnetic canting, as shown in Figure 1.

The magnetic *Pnm′a* symmetry of the ground state allows for various ME multipoles at the Ni sites, which have magnetic point group symmetry *mm′m*. These ME multipoles in turn have either ferro or anti-ferro type arrangements, as was pointed out previously in Ref. 2. In particular, the symmetry allows for toroidal moments along the *y* direction ( *t _y_*) and *q _xz_* quadrupole moment components, both with ferro-type arrangements, leading to a net *t _y_* and *q _xz_* in the system. In addition, the ME monopole *a* and the *q*_*x* ^2^− *y* ^2^_, *q*_*z* ^2^_ quadrupole moments are also allowed at the Ni sites. These multipoles have opposite signs at the neighboring Ni sites, however, leading to net zero contributions to *a*, *q*_*x* ^2^− *y* ^2^_, and *q*_*z* ^2^_. Our density functional results for the ME multipoles are consistent with this symmetry analysis. The computed magnitudes of atomic-site contributions to the various allowed ME multipoles, and their relative orientations at different Ni sites are listed in Table II. We note that the computed magnetic moment and the ME multipole moment values depend on the choice of atomic radius. However, this does not affect any physical properties, such as magnetization density or the corresponding electron momentum density. The computed magnetic moment also depends on the value of Hubbard *U*. However, the antisymmetric part of the Compton profile has only a weak dependence on *U*, which is likely related to the fact that, unlike magnetic moment, the *U* parameter does not have any significant impact on the antisymmetric part of the magnetization density ^24^. As our calculation shows the presence of a net toroidal moment along the *y* direction, we expect an antisymmetric Compton profile along the same direction in momentum space ^15^, which we now proceed to discuss.

**Figure 3.**
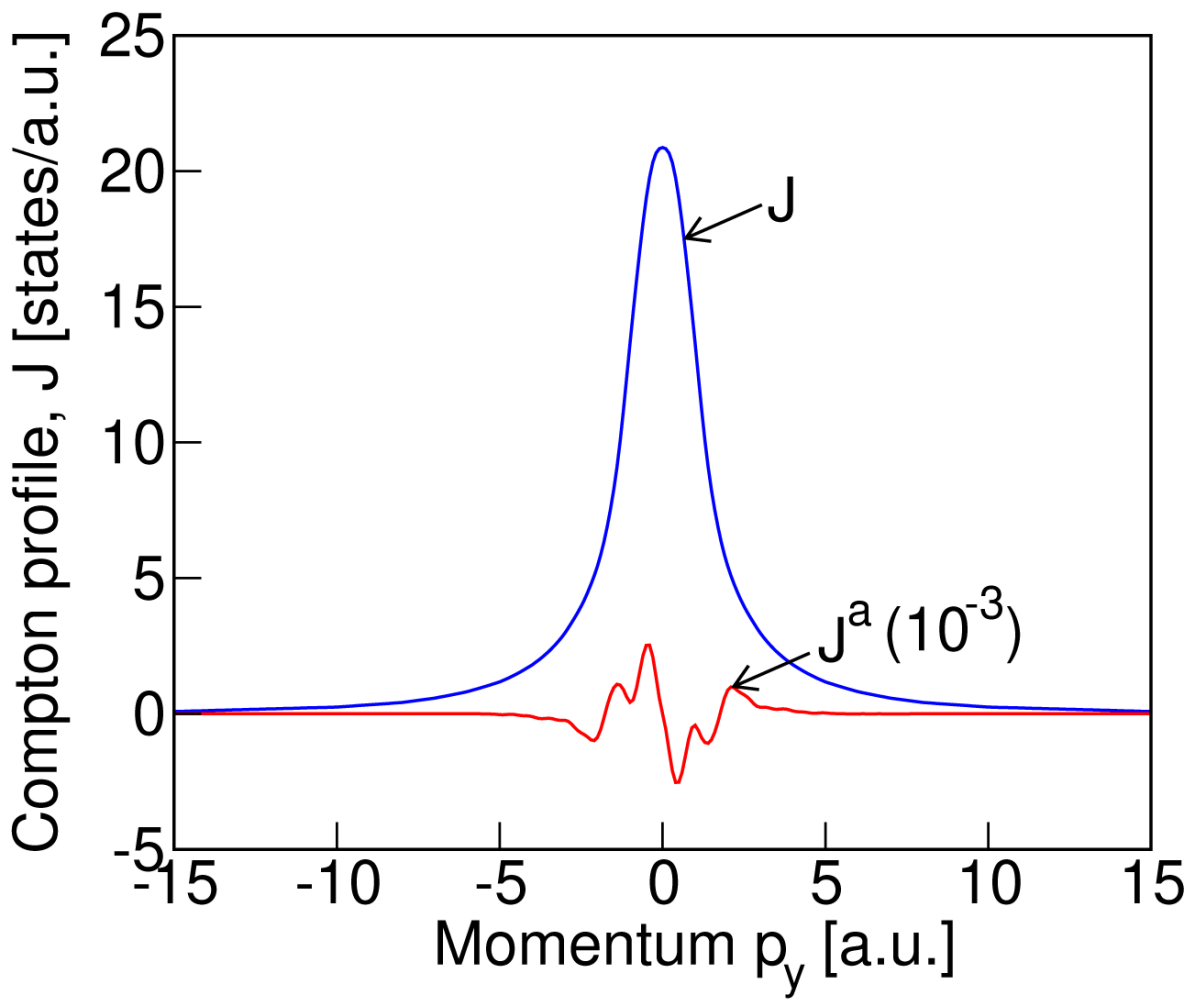
The computed (without convolution) total, *J*, and antisymmetric, *J ^a^*, Compton profile for the *Pnm′a* magnetic structure (see Figure 1 for the spin arrangement) of LiNiPO _4_.

We compute the Compton profile in the magnetic ground state of LiNiPO _4_ along the three Cartesian directions. In agreement with the expectation from symmetry arguments, we find an antisymmetric component in the Compton profile only along the *y* direction, which is the direction of the toroidal moment *t _y_* , in momentum space. Our calculated total Compton profile *J* and the antisymmetric part, *J ^a^*, are depicted in Figure 3. Note that the antisymmetric contribution has been multiplied by 10 ^3^. We can see from this figure that the antisymmetric part of the profile is approximately four orders of magnitude smaller than the corresponding symmetric part of the profile. Although small, this signal should be at the limit of detectability, motivating the explicit measurements on LiNiPO _4_, which we present in Section IV B.

Finally for this section, we analyze both parts of the profile to verify the zero-sum rules

$$\int_{-\infty }^{\infty }p_{y}J^{s,a}(p_{y})dp_{y}=0\;.\;(3)$$

This is a trivial condition for the symmetric part *J ^s^* and is always satisfied because the integrand in Equation 3 is by defnition always an odd function in *p _y_*, resulting in a zero value upon integration. In contrast, for the antisymmetric part, *J ^a^*, the zero-sum rule imposes strict conditions on the positive- *p _y_* and negative- *p _y_* halves of the profile separately ^15^, with

$$\int_{-\infty }^{0}p_{y}J^{a}(p_{y})dp_{y}=\int_{0}^{\infty }p_{y}J^{a}(p_{y})dp_{y}=0\;.\;(4)$$

We find that our computed antisymmetric Compton profile satisfies this condition to threee decimal places.

### B. Experimental results and comparison with theory

**Figure 4.**
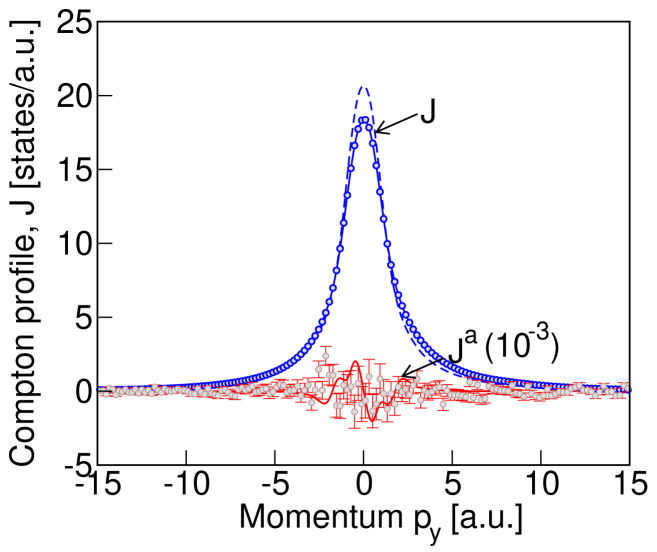
The measured Compton profile for LiNiPO _4_ compared with the calculated profile of Figure 3. The measured total Compton profile is shown by the blue circle data points and the blue solid line, while the measured antisymmetric profile, magnified by a factor of 10 ^3^, is indicated by the red circle data points and red vertical line error bars. The convoluted computed profiles with a Gaussian of FWHM = 0.44 from Figure 3 are shown as the blue dashed line (total profile) and red solid line (antisymmetric part of the calculated profile, magnified by a factor of 10 ^3^). While the peak in computed total profile differs from the measured value by about 10% (see text for explanation), the measured and computed antisymmetric profiles are of the same orders of magnitude. The data shown here represent approximately 42 hours data collection, obtained over four days of measurement.

In Figure 4 we show our measured total Compton profile (blue solid line with blue circles as data points) and the antisymmetric signal (red circles with error bars, multiplied by 10 ^3^). The total profile is normalized so that the integral of the profile gives the total number of electrons in the unit cell of LiNiPO _4_ with four formula units. The antisymmetric profile is derived from the difference in Compton profiles measured in the presence of magnetic fields with opposite directions (see Equation 2). For comparison, the calculated profiles of Figure 3, after convolution with a Gaussian of full width at half maximum equal to our experimental resolution of 0.44 a.u., are also shown in Figure 4. The blue dashed line and red solid line depict the calculated convoluted total and antisymmetric profiles respectively. As seen from Figure 4, the peak in the calculated total profile is about 10% higher than the measured value. We also notice the skewness of the measured peak in the total Compton profile. We attribute both the skewness and the difference in measured and computed total profiles to multiple scattering effects, which are not considered here. The reason for ignoring the multiple photon scattering is that it does not have any significant contribution to the measured difference signal that was obtained by subtracting the data measured by reversing the magnetoelectric domains, as discussed in section III C. This is because although the multiple scattering contributes to the total Compton profile, when we take the difference in the data sets, measured with and without reversing the magnetoelectric domain, the multiple scattering contribution largely cancels out. Interestingly, we see that the data points corresponding to the measured antisymmetric signal are consistent with our calculated antisymmetric Compton profile, both being around four orders of magnitude smaller than the total profile. Frustratingly, however, both are at the level of the experimental noise (indicated by red error bars in Figure 4), and we are not able to conclusively infer the existence of a non-zero antisymmetric contribution. Our analysis suggests that the experiment came very close to measuring a signal of the calculated magnitude and that a modest increase in experimental signal to noise ratio would likely have yielded a positive result.

## V. Summary

To summarize, we have studied the Compton profile in LiNiPO _4_, the magnetic ground state of which allows for a net toroidal moment *t _y_*. Our density functional calculations show the existence of an antisymmetric component in the Compton profile of LiNiPO _4_ along the same *y* direction in the momentum space as the toroidal moment, implicating antisymmetric Compton scattering as a possible signature of a time-odd, parity-odd ME toroidal moment *t _y_* in the material. The calculated magnitude of the computed antisymmetric component is small, however, with magnitude ~ 10 ^−4^ times the calculated total Compton profile.

Our Compton scattering measurements on LiNiPO _4_ also find a weak difference signal, consistent with the computed order of magnitude. Unfortunately, however, the weak signal is of the same order of magnitude as the statistical error, preventing us from conclusively determining an antisymmetric profile.

Our finding that the predicted and measured antisymmetric Compton response of LiNiPO _4_ is exactly at the noise level motivates further research in two directions: First, materials with a larger antisymmetric Compton profile should be identified, so that existing experiments will provide an unambiguous signal. Second, since the experimental signal to noise ratio was limited by the detector count-rate capability, solid angle, incident beam flux and monochromator bandwidth, optimization of these factors, while practically challenging, could improve the signal to noise by an order of magnitude, allowing anti-symmetric Compton profiles to be determined with ease.

## Data availability

### Underlying data

The experimental data is available as follows:

Zenodo: Anti-symmetric Compton scattering in LiNiPO _4_: Towards a direct probe of the magneto-electric multipole moment. https://doi.org/10.5281/zenodo.5167599 ^25^

This project contains the following experimental files:

- Data file description.docx: Description of data files
- data.zip: Experimental data files
- photos.zip: Photographs from the experiment
- evernote log electronic logbook.pdf: Electronic logbook for the experiment
- SPring-8 antisymmetric compton proposal may 2017.pdf: Beamtime proposal

The theoretical data is available as follows:

Materials Cloud: Anti-symmetric Compton scattering in LiNiPO _4_: Towards a direct probe of the magneto-electric multipole moment. https://doi.org/10.24435/materialscloud:yx-7k ^26^

The description of the files and folders for this project is the following:

- The main folder “data” contains the two separate folders, “3 × 6 × 6” and “5 × 10 × 10” corresponding to the two different k-grids, used for the theoretical calculations of the Compton profile in LiNiPO _4_. Each folder contains all the input files necessary for reproducing our results, and the output data files for the theoretical Compton profile, that are presented in the paper. The folder “3 × 6 × 6” contains the sub-folder “fig3-data-files” that includes the explicit data files corresponding to the theoretical plots in Figure 3 of the paper.

Data are available under the terms of the Creative Commons Attribution 4.0 International license (CC-BY 4.0).
