# Supplementary material for: Anti-symmetric Compton scattering in LiNiPO 4: Towards a direct probe of the magneto-electric multipole moment
Source: Open Res Eur. 2022 May 5;1:132. Originally published 2021 Nov 1. [Version 2] doi: 10.12688/openreseurope.13863.2 (PMC10446042; doi:10.12688/openreseurope.13863.2)
Supplement: Peer review history

# Peer review: Reviewer response for version 2

**DOI:** 10.21956/openreseurope.15909.r29169

**Published:** 2022-05-12

**Authors:** Niels Christensen

**Affiliations:** 1 Department of Physics, Technical University of Denmark, Kongens Lyngby, Denmark

The reply letter and the updated manuscript fully address my initial slight reservations. I am happy to support the indexing of this manuscript.

Is the study design appropriate and does the work have academic merit?

Yes

Is the work clearly and accurately presented and does it cite the current literature?

Yes

If applicable, is the statistical analysis and its interpretation appropriate?

Yes

Are all the source data underlying the results available to ensure full reproducibility?

Yes

Are the conclusions drawn adequately supported by the results?

Yes

Are sufficient details of methods and analysis provided to allow replication by others?

Partly

Reviewer Expertise:

I am an experimental physicist studying magnetic order and fluctuations using neutron scattering. In connection with the present manuscript my expertise is related to having performed numerous neutron scattering experiments on LiNiPO4 and related Lithium orthophosphates.

I confirm that I have read this submission and believe that I have an appropriate level of expertise to confirm that it is of an acceptable scientific standard.

# Response

**Published:** 2022-05-17

**Authors:** Sayantika Bhowal

**Affiliations:** ETH, Zurich, Switzerland

We thank the referee for the positive feedback and for recommending the indexing of the manuscript.

# Peer review: Reviewer response for version 2

**DOI:** 10.21956/openreseurope.15909.r29168

**Published:** 2022-05-09

**Authors:** Hiroshi Sakurai

**Affiliations:** 1 Faculty of Science and Technology, Gunma University, Kiryu, Japan

The authors explained the measurement time. The situation can be understood.  The manuscript can pass peer review as is.

Is the study design appropriate and does the work have academic merit?

Yes

Is the work clearly and accurately presented and does it cite the current literature?

Yes

If applicable, is the statistical analysis and its interpretation appropriate?

Partly

Are all the source data underlying the results available to ensure full reproducibility?

Yes

Are the conclusions drawn adequately supported by the results?

Yes

Are sufficient details of methods and analysis provided to allow replication by others?

Yes

Reviewer Expertise:

NA

I confirm that I have read this submission and believe that I have an appropriate level of expertise to confirm that it is of an acceptable scientific standard.

# Peer review: Reviewer response for version 1

**DOI:** 10.21956/openreseurope.14947.r27943

**Published:** 2021-12-07

**Authors:** Niels Christensen

**Affiliations:** 1 Department of Physics, Technical University of Denmark, Kongens Lyngby, Denmark

This manuscript reports electronic structure calculations performed within density functional theory and Compton scattering experiments aimed to detect the anti-symmetric contribution to the electronic momentum distribution caused by the existence of a toroidal moment in LiNiPO4.

 The experimentally detected anti-symmetric contribution is of the same order of magnitude (relative to the symmetric part) as predicted by theory. However, the observed signal is also comparable to the experimental noise, prohibiting a conclusive demonstration of the ability of Compton scattering to detect the presence of a toroidal moment.

 The manuscript is interesting and well-written. I have only a few minor comments:

 1. Given that the magnitude of the predicted asymmetric contribution to the momentum distribution is 4 orders of magnitude lower than the symmetric part, I wonder how accurate the DFT calculations are, in particular since they predict a magnetically ordered moment of approximately 1.69 Bohr magnetons (Table II) where experiments have found approximately 2.2 Bohr magnetons (Reference 17). It is clear that such a discrepancy in the magnetic moments does not prohibit the existence of a toroidal moment and an associated antisymmetric contribution to the momentum distribution, but it could affect the magnitude of the effect. I wonder if the authors would comment on this.

 2. The description of the field-cooling protocol is potentially slightly confusing (In particular around the sentences "We were therefore ... one hour per cycle"). Could the authors please clarify whether or not the magnetic field was ramped inside or outside of the magnetically ordered state during their experiment?

 3. A clear indication of the counting time required to obtain the experimental data would be useful for anyone attempting to replicate these experiments.

 4. Just below Eq. (3), "defnition" should be "definition".

Is the study design appropriate and does the work have academic merit?

Yes

Is the work clearly and accurately presented and does it cite the current literature?

Yes

If applicable, is the statistical analysis and its interpretation appropriate?

Yes

Are all the source data underlying the results available to ensure full reproducibility?

Yes

Are the conclusions drawn adequately supported by the results?

Yes

Are sufficient details of methods and analysis provided to allow replication by others?

Partly

Reviewer Expertise:

I am an experimental physicist studying magnetic order and fluctuations using neutron scattering. In connection with the present manuscript my expertise is related to having performed numerous neutron scattering experiments on LiNiPO4 and related Lithium orthophosphates.

I confirm that I have read this submission and believe that I have an appropriate level of expertise to confirm that it is of an acceptable scientific standard, however I have significant reservations, as outlined above.

# Response

**Published:** 2022-04-19

**Authors:** Sayantika Bhowal

**Affiliations:** ETH, Zurich, Switzerland

We thank the Referee for critically reading our paper and for finding the manuscript interesting. Below we address the comments of the Referee:

1. In the DFT calculation, the value of the magnetic moment (toroidal moment) is obtained by projecting the magnetization density (antisymmetric part of the magnetization density) into an atomic sphere of the Ni ion. As a result, the explicit value of computed Ni magnetic moment (toroidal moment) depends on the choice of ionic radius of Ni. The difference from the experimentally reported magnetic moment may be attributed to this choice of radius, which, however, does not affect the physical property of the system such as magnetization density (antisymmetric part of the magnetization density) and the corresponding antisymmetric electron density in the momentum space and, hence, the antisymmetric Compton profile.
  Another factor that can lead to deviation from the experimentally measured magnetic moment value is the choice of Hubbard *U* in the DFT calculation. However, as shown in Ref. Phys. Rev. Research **3**, 033185 (2021), the antisymmetric part of the Compton profile has only a weak dependence on the choice of Hubbard *U*. This is because although Hubbard *U* enhances the Ni magnetic moment, the anti-symmetric part of the magnetization density (which determines the toroidal moment in the system) remains more or less unaffected. Thus it is likely that these effects will not affect the magnitude of the antisymmetric Compton profile.
  A couple of sentences, starting with "We note that....", and Ref. 24 are added in section IV-A of the revised version.
2. The sample temperature was raised to around 30K (above T _c_) before each time the field was ramped. The text in section III-C has been updated to clarify this.
3. The data in the figure represents approximately four days of data acquisition. The Fig. 4 caption has been updated to reflect this.
4. The typo is corrected in the revised version.

# Peer review: Reviewer response for version 1

**DOI:** 10.21956/openreseurope.14947.r28011

**Published:** 2021-12-06

**Authors:** Hiroshi Sakurai

**Affiliations:** 1 Faculty of Science and Technology, Gunma University, Kiryu, Japan

The authors tried to catch the magnetoelectric toroidal moments in LiNiPO4 experimentally by measuring Compton scattering effects. This research seems to be important for fundamental physics of the magnetoelectric multipoles and the possible technological applications of magnetoelectric materials. Understanding as well as detecting such magnetoelectric multipoles is becoming an active area of research in condensed matter physics.

 A simple question. How long does it take to get Figure 4? Is it enough for the statistical accuracy?

 I think the most important point of this article is to prove the observation of topological electronic structure in momentum space. The theoretical prediction seems to be valid. However it is not enough to prove it by the experiment because the  statistical accuracy is not enough. If the authors measure longer, there is a possibility to succeed. A reader may want to know how long it takes to get Figure 4. (I was not able to catch it). 

 The author may describe it in the experimental section.

Is the study design appropriate and does the work have academic merit?

Yes

Is the work clearly and accurately presented and does it cite the current literature?

Yes

If applicable, is the statistical analysis and its interpretation appropriate?

Partly

Are all the source data underlying the results available to ensure full reproducibility?

Yes

Are the conclusions drawn adequately supported by the results?

Yes

Are sufficient details of methods and analysis provided to allow replication by others?

Yes

Reviewer Expertise:

NA

I confirm that I have read this submission and believe that I have an appropriate level of expertise to confirm that it is of an acceptable scientific standard, however I have significant reservations, as outlined above.

# Response

**Published:** 2022-04-19

**Authors:** Sayantika Bhowal

**Affiliations:** ETH, Zurich, Switzerland

We thank the Referee for critically reading the article and for the valuable suggestion. The data represented in Fig. 4 took approximately four days to collect. Significant improvements in statistical quality would not be practical through longer acquisition alone, but would require changes to the experimental setup and detector systems, as discussed.

 Following the suggestion of the Referee, we have added a sentence to Fig. 4 caption in the revised manuscript.

# Peer review: Reviewer response for version 1

**DOI:** 10.21956/openreseurope.14947.r27944

**Published:** 2021-11-22

**Authors:** Lars Nordstrom

**Affiliations:** 1 Department of Physics and Astronomy, Uppsala University, Uppsala, Sweden

- This report is on a combined theoretical and experimental study on the anti-symmetric Compton scattering on a magneto-electric material LiNiPO4 in the strive to observe local toroidal moments.
- As is clearly stated in the manuscript the conclusions are not strictly conclusive as the experimental error bars are of the same order as the variations in the detected signal. However, it is on the other hand not contradicting the theoretical study.
- The report outlines the way to proceed in future studies to study local magnet-electric moments where the experimental resolution can be increased.
- This study is sound and honest and deserves indexing.

Is the study design appropriate and does the work have academic merit?

Yes

Is the work clearly and accurately presented and does it cite the current literature?

Yes

If applicable, is the statistical analysis and its interpretation appropriate?

Yes

Are all the source data underlying the results available to ensure full reproducibility?

Yes

Are the conclusions drawn adequately supported by the results?

Yes

Are sufficient details of methods and analysis provided to allow replication by others?

Yes

Reviewer Expertise:

My expertise is corresponding to the theoretical side of this study. Especially regarding the local multipoles of magnetically ordered materials, not least in systems which are both correlated and relativistic.

I confirm that I have read this submission and believe that I have an appropriate level of expertise to confirm that it is of an acceptable scientific standard.

# Response

**Published:** 2022-04-19

**Authors:** Sayantika Bhowal

**Affiliations:** ETH, Zurich, Switzerland

We thank the Referee for positive feedback and for recommending publication of the manuscript.
